# Functional Analysis of a Novel CLN5 Mutation Identified in a Patient With Neuronal Ceroid Lipofuscinosis

**DOI:** 10.3389/fgene.2020.536221

**Published:** 2020-09-02

**Authors:** Sukun Luo, Bo Bi, Baiqi Zhu, Li Tan, Peiwei Zhao, Yufeng Huang, Gefei Wu, Aifeng Zhou, Xuelian He

**Affiliations:** ^1^Precision Medical Center, Wuhan Children’s Hospital (Wuhan Maternal and Child Healthcare Hospital), Tongji Medical College, Huazhong University of Science and Technology, Wuhan, China; ^2^Rehabilitation Department, Wuhan Children’s Hospital (Wuhan Maternal and Child Healthcare Hospital), Tongji Medical College, Huazhong University of Science and Technology, Wuhan, China; ^3^Department of CT & MRI, Wuhan Children’s Hospital (Wuhan Maternal and Child Healthcare Hospital), Tongji Medical College, Huazhong University of Science and Technology, Wuhan, China; ^4^Neurology Department, Wuhan Children’s Hospital (Wuhan Maternal and Child Healthcare Hospital), Tongji Medical College, Huazhong University of Science and Technology, Wuhan, China

**Keywords:** neuronal ceroid lipofuscinoses, *CLN5*, cellular localization, whole exome sequencing, cellular trafficking

## Abstract

Neuronal ceroid lipofuscinoses (NCLs) are a group of autosomal recessive inherited neurodegenerative disorders mainly affecting children, and at least 13 causative genes (*CLN1* to *CLN8* and *CLN10* to *CLN14*) have been identified. Here, we reported a novel homozygous missense mutation (c.434G > C, p.Arg145Pro) identified in *CLN5* gene via whole exome sequencing in a 5-year-old girl. The patient first presented paroxysmal epilepsy associated with vomiting, followed by progressive regression in walking, vision, intelligence and speaking. Combining the molecular and clinical analysis, the diagnosis of NCL could be made, although the missense mutation (c.434G > C, p.Arg145Pro) in CLN5 was evaluated to be a variant of uncertain significance according to American College of Medical Genetics and Genomics (ACMG) standard. We further performed expression and localization studies and our results provide evidence of impaired cellular trafficking of CLN5 to lysosome, indicating that this mutation might be deleterious to the function of CLN5 for its mislocalization. Our study demonstrated the efficacy of next generation sequencing in molecular diagnosis, and a deleterious effect of the variant discovered in our patient on CLN5, triggering the NCL disease.

## Introduction

Neuronal ceroid lipofuscinoses (NCLs) are a heterogeneous group of progressive neurodegenerative disorders, and are relatively rare diseases with an estimate incidence ranging from 1 to 4 per 100,000 worldwide (Simonati et al., 2017). It is characterized by epilepsy, difficulties in learning, progressive visual loss, motor deterioration, cognitive decline, and a reduced lifespan. The age of clinical onset of NCLs ranges from infancy to adulthood. Pathologically, these disorders share a common hallmark of accumulation of autofluorescent storage material resembling ceroid and lipofuscin in lysosomes in neural and peripheral tissues. Except for the only clinically-approved enzyme replacement for CLN2 disease, one subtype of NCLs, no curative treatment is available for other subtypes (Mole et al., 2018). Although the precise underlying mechanisms remain elusive, at least 13 pathogenic genes (*CLN1* to *CLN8* and *CLN10* to *CLN14*) have been identified to be linked to NCLs. The NCL caused by *CLN5* mutations is a rare subtype (OMIM 256731), first described in Finland and later identified in a variety of ethnic groups from both eastern and western countries (Savukoski et al., 1998; Xin et al., 2010). Generally, patients caused by *CLN5* mutations have an average age of onset between 4 and 7 years, but some manifest clinical symptoms can start as early as 2 years or late at their adulthood (Mancini et al., 2015). The *CLN5* gene is located on chromosome 13q21-q32 and encodes a 407-amino acid glycoprotein with a molecular mass of 52∼75kD (Isosomppi et al., 2002; Schmiedt et al., 2010). CLN5 is initially translated as a type II transmembrane protein with heavy glycosylation, subsequently cleaved into a mature soluble protein and resides in lysosome compartment (Isosomppi et al., 2002; Jules et al., 2017). As previously reported, CLN5 does not share homology with any known proteins, is highly expressed in the brain and is associated with brain development (Holmberg et al., 2004).

To date, more than 50 *CLN5* mutations are collected in the HGMD database^1^, and less than 10 mutations have been reported in Chinese populations, including c.334C > T (p.Arg112Cys), c.595C > T (p.Arg199^∗^), c.620G > C (p.Trp207Ser), c.623G > A (p.Cys208Tyr), c.718_719delAT (p.Met240Valfs^∗^13), c.1071_1072delCT (p.Leu358Alafs^∗^4), c.1082T > C (p.Phe361Ser), c.1100-1103delAACA (p.Lys368 Serfs^∗^15), and c.321-1G > A intron splice site, and there is no hotspot mutation (Xin et al., 2010; Ren et al., 2016, 2019; Ge et al., 2018; Zhou et al., 2018). In this study, we identified and characterized a novel missense mutation c.434G > C (p.Arg145Pro) in *CLN5* in a suspected NCL patient by using next generation sequencing. Our experiment results demonstrated that this mutation did not affect the protein expression but impaired the subcellular trafficking of CLN5 to lysosome, suggesting that it should be pathogenic for its mislocalization. Our study expands mutation spectrum in *CLN5*, as well as provides the clinical features and functional consequences of a novel mutation.

## Materials and Methods

### Case Presentation

**FIGURE 1:**
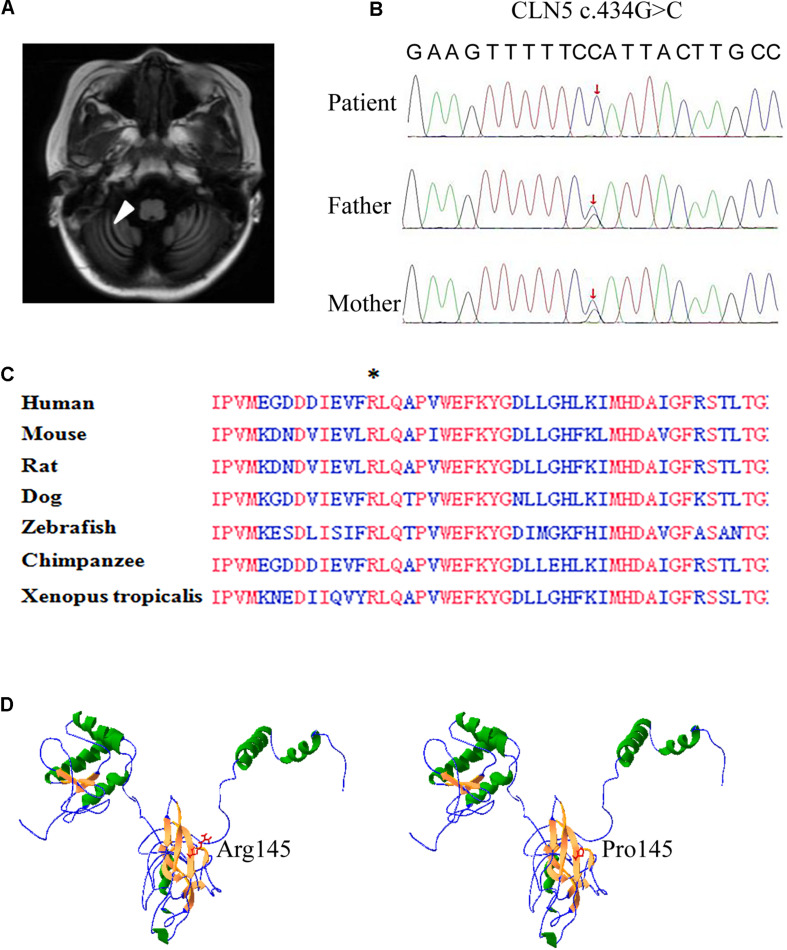
Brain MRI and whole exome sequencing findings and *in silico* prediction of Arg145. **(A)** Axial and coronary T2 weighted brain resonance magnetic images of the patient revealed cerebellar atrophy. **(B)** Confirmation of the missense mutation (c.434G > C) in the genomic DNA of the patient and her parents. Sanger sequencing showed the patient was homozygous for the mutation and her parents were heterozygous carriers, as labeled by red arrow. **(C)** Alignment of partial CLN5 amino acid sequence in 7 vertebrate species. CLN5 shares a highly homology among different species and the absolutely identical residues are labeled by red. An asterisk indicates arginine 145th (the mutant amino acid of c. 434G > C) found in patient. **(D)** Structure model of CLN5 predicted by RaptorX. Arginine of residue 145th was located on a β-sheet, which was composed of Gly140∼Met146. Substitution of arginine by proline greatly interrupted the interaction of residue 145th and other nearby amino acids in space.

An 8-year-old girl presented with psychomotor and visual deterioration, which began at the age of 5. She was the first and only child of a healthy non-consanguineous Chinese couple. She was born full-term with a birth weight of 3.8 kg, and had unremarkable prenatal, neonatal and family history. Before 5 years old, she did not show obvious abnormalities in psychomotor development and vision. Paroxysmal epilepsy associated with vomiting was first noticed at the age of 5.5 years. Electroencephalogram displayed lots of delta rhythm, diffuse generalized slow spike and slow wave, and brain MRI showed prominent fissures or sulci on cerebellar hemisphere as arrow pointed, indicating cerebellar atrophy (Figure 1A). The seizures were controlled temporarily by sodium valproate. However, her neurologic condition gradually worsened and epilepsy frequently recurred after 1 year. Subsequently, she developed progressive ataxia, regression of speech, and vision decline. She completely lost her vision and walking ability as well as vocalization within 2 years.

### Mutation Analysis

After obtaining written informed consent from her parents and ethical approval from Wuhan Children’s Hospital Ethics Committee, genomic DNA was extracted from whole blood of the patient and her parents. Whole exome sequencing was performed on Illumina HiSeq2000 according to the manufacturer’s instructions. The identified sequence variant of interest was confirmed in the patient and her parents by Sanger sequencing. Polyphen-2^2^, SIFT^3^, MutationTaster^4^, and CADD^5^ were used to predict the potential effect of the Arg145Pro on function and structure of CLN5 protein. We also predicted its effect on the three-dimensional structure of CLN5 by using RaptorX^6^ (Kallberg et al., 2012).

### Cells, Plasmids, and Antibodies

Hela cells were grown in DMEM supplemented with 10% fetal bovine serum (Gibco). The plasmids encoding wild-type CLN5-HA (defined as wtCLN5) were purchased from GeneCopoeia. The CLN5 Arg145Pro mutant (defined as mtCLN5) was constructed by site-directed mutagenesis and confirmed by DNA sequencing. Mouse anti-HA tag IgG (sc-7392) and monoclonal antibody against CLN5 (sc-374672) were bought from Santa Cruz, while rabbit polyclonal antibody against LC3I/II(4108S), p62/SQSTM1 rabbit monoclonal antibody (8025S), LAMP-1 rabbit monoclonal antibody (9091T) and HRP-linked anti-rabbit IgG (7074S) were purchased from CST. HRP-linked anti-mouse IgG, mouse monoclonal antibody against GAPDH, Alexa Fluor 488-conjugated goat anti-rabbit IgG and Alexa Fluor 564-conjugated goat anti-mouse IgG were purchased from Boster.

### Western Blot

HeLa cells pre-seeded on six-well plate were transfected with 2.5μg plasmids encoding wtCLN5-HA or mtCLN5-HA per-well by using Lipofectamine 2000 (Invitrogen). Twenty four hours post-transfection, cells were lysed with lysis buffer in the presence of protease inhibitor cocktail (Roche). Protein samples were resolved by 12% SDS-PAGE and transferred onto PVDF membrane. After being blocked with 3% BSA in TBS-T, the PVDF membrane was subsequently incubated with mouse anti-HA tag IgG or antibody against CLN5 or mouse monoclonal antibody against GAPDH and HRP-linked anti-mouse IgG. And then immunoreactive bands were visualized with ECL chemiluminescent substrate (ThermoFisher). The expression levels of autophagy related proteins LC3I/II, LAMP-1 and p62/SQSTM1 were detected by using corresponding antibodies as described above.

### Immunofluorescence and Confocal Microscopy

A total of 8 × 10^4^ HeLa cells were seeded on 35 mm glass bottom culture dishes and transfected with 1μg plasmids encoding wtCLN5 or mtCLN5 per-well. Twenty four hours later, transfected cells were fixed in 4% paraformaldehyde and permeabilized with 0.2% Triton-X100 in PBS both for 15 min at room temperature. After three washes with PBS, cells were blocked by 3% BSA in PBS and then incubated with a mouse anti-HA tag IgG and a rabbit monoclonal antibody against LAMP-1 both at a dilution of 1:100 for 1 h at room temperature. Following three washes, cells were subsequently incubated with Alexa Fluor 488-conjugated goat anti-rabbit IgG and Alexa Fluor 564-conjugated goat anti-mouse IgG at a dilution of 1:200 for 1 h at room temperature. Cell nuclei were stained with DAPI. After extensive washes, stained cells were analyzed under Nikon A1 MP confocal microscope equipped with a 60 × oil immersion objective. Image sequences were processed and analyzed using NIS-elements Viewer software (Nikon).

### Cycloheximide Chase Assay

HeLa cells pre-seeded on 35 mm glass bottom culture dishes were transfected with plasmids encoding wtCLN5 or mtCLN5 by using Lipofectamine 2000 (Invitrogen). 24 h post-transfection, the transfected cells were incubated with 50μM cycloheximide (MCE), a protein synthesis inhibitor, for 5 h (Isosomppi et al., 2002). Thereafter, the cells were fixed for confocal microscopy assay as described above.

## Results

### Mutation Analysis

Whole exome sequencing was performed on the patient and her parents. A mutation (c.434G > C, p. Arg145Pro) in the exon 2 of *CLN5* gene was identified in this patient and confirmed by Sanger sequencing to be homozygous in the proband and heterozygous in her parents (Figure 1B).

The c. 434G > C in *CLN5* was a novel missense mutation and was not collected in the HGMD, NCL mutation database^7^, and any other online database. *In silico* prediction indicated that CLN5 is highly conserved during evolution and the arginine at the 145th residue is identical among 7 vertebrate species (Figure 1C), implying this conserved residue might be critical for CLN5 function. The missense mutation was predicted to be probably tolerated by SIFT with score 0.084, deleterious by PROVEAN with score −4.02, probably damaging by Polyphen-2 with score 1.00, disease-causing by MutationTaster with score 1.00 and damaging by CADD with score 26.9. To test if Arg145Pro affect the 3D structure of CLN5, the 3D structure of CLN5 was predicted by using RaptorX and the location of Arg145 was labeled. As shown in Figure 1D, Arg145 resided on a β-sheet, consisting of Asp140∼Lys146 and the change of arginine to proline greatly interrupted the interaction of R145 and other nearby amino acids in space. According to ACMG guideline, the missense mutation c.434G > C was predicted to be a variant of uncertain significance. Therefore, the pathological significance of this variant remains to be further clarified.

### Functional Studies of Arg145Pro

**FIGURE 2:**
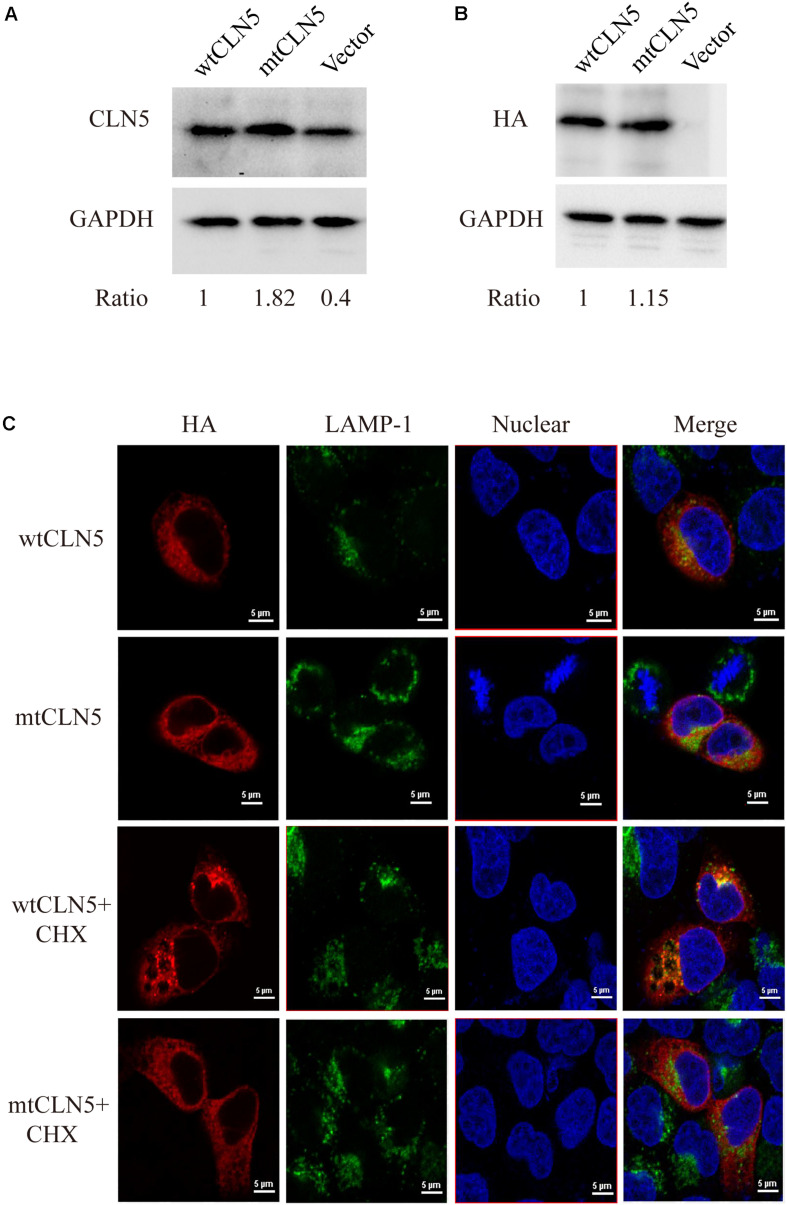
Functional analysis of p.Arg145Pro. **(A,B)** Impacts of Arg145Pro on the expression of CLN5. HeLa cells were transfected with wtCLN5 or mtCLN5 and total expression of CLN5 was detected by Western blot using antibody against CLN5 or anti-HA tag IgG with GAPDH as a loading control. One representative experiment out of three is shown. **(C)** Impacts of Arg145Pro on the cellular localization of CLN5. Cells transfected with wtCLN5 or mtCLN5 were treated with 50μM cycloheximide for 5 h and then fixed with 4% paraformaldehyde and subsequently co-stained with anti-HA tag antibody (red) and antibody against LAMP-1 specifically recognizing lysosome (green). Co-localization of CLN5 and lysosome was analyzed by confocal microscopy. Transfected cells with no treatment are control. Representative confocal images from three independent experiments are shown.

Plasmids encoding wtCLN5 or mtCLN5 were transfected into Hela cells, 24 h later these transfected cells were lysed or fixed for following gel analyses or confocal microscopy. As shown in Figures 2A,B, a CLN5 polypeptide band about 50kD was detected in both wtCLN5 and mtCLN5 transfected cells by antibody against CLN5 or HA, respectively. However, it was noticeable that the relative intensity of the polypeptide bands was different between the two transfected cells according to the difference of detected antibodies. The HA antibody, binding transiently expressed polypeptides, detected a comparable expression level of wtCLN5 and mtCLN5 (Figure 2B), implying that Arg145Pro might not affect CLN5 protein expression. In Figure 2A, expression of total CLN5 polypeptide was analyzed and the mtCLN5 transfected cells displayed a higher expression level than that of wild-type group. As reported previously, mature CLN5 was mainly targeted to lysosome, but some would be secreted out of cell (Larkin et al., 2013). Therefore, we hypothesized that overexpression of mtCLN5 might alter the basic expression of CLN5 in HeLa cells or that Arg145Pro might impair the trafficking and secretion of mtCLN5, which resulted in an increase of total CLN5 polypeptide in mtCLN5 transfected cells.

CLN5 was a lysosomal glycoprotein and it should be correctly targeted to lysosome for normal function. Since we proposed that Arg145Pro might exert its influence by interrupting the intracellular transport of CLN5 protein, we next investigated the co-localization of wtCLN5 or mtCLN5 with LAMP-1, a lysosomal marker, to test this hypothesis. As shown in Figure 2C, both overexpressed wtCLN5 and mtCLN5 were distributed around the nucleus and mildly co-localized with LAMP-1 in transfected cells, but in cells treated with the translation inhibitor cycloheximide for 5 h, wtCLN5 was gathered and showed an increased co-localization with LAMP-1, whereas mtCLN5 did not co-localize with LAMP-1. All these results suggested that Arg145Pro impaired the intracellular trafficking of CLN5, with little mtCLN5 entering into lysosome. These findings collectively indicated that Arg145Pro had no effects on the expression of CLN5, but affected its subcellular localization, which might harm the normal functions of CLN5.

**FIGURE 3:**
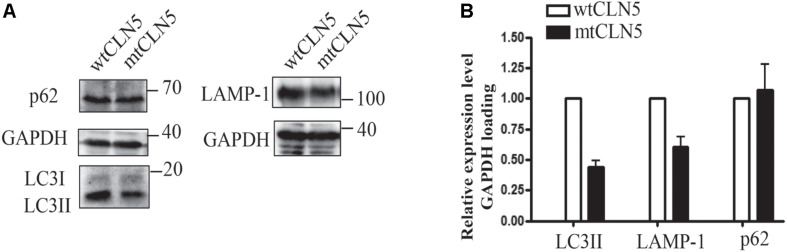
Impacts of Arg145Pro on the expression of autophagy related proteins. **(A)** HeLa cells were transfected with wtCLN5 or mtCLN5, and the expression levels of autophagy related proteins LC3II, LAMP-1 and p62/SQSTM1 were detected by Western blot using corresponding antibodies with GAPDH as a loading control. One representative experiment out of three is shown. **(B)** Relative intensity of GAPDH-loaded autophagy related proteins in wtCLN5 and mtCLN5 transfected cells from 3 independent experiments. Gray scale scanning was performed by Image J software and the relative expression level of LC3II, LAMP-1 and p62/SQSTM1 in mtCLN5 transfected cells was normalized by that in wtCLN5 transfected cells.

Since most of mtCLN5 was not correctly targeted to lysosome, we next investigated the impact of Arg145Pro on the function of CLN5. Given that the precise function of CLN5 was not well-defined and that autophagy disruption was indicated to be associated with NCL pathogenesis (Seranova et al., 2017), we investigated the impacts of mtCLN5 on autophagy. Microtubule-associated protein light chain 3(LC3) is a marker of autophagy, and the increase in the conversion from its cytosolic form (LC3-I) to the membrane-bound form (LC3-II) indicates increased formation of autophagosomes, suggesting increased autophagy (Lee and Lee, 2016). While LAMP-1 is essential for phagosome-lysosome fusion (Huynh et al., 2007). As shown in Figures 3A,B, LC3-II and LAMP-1 slightly decreased in mtCLN5 transfected cells, indicating that mtCLN5 might be deleterious to autophagy of the transfected cells. Taken together, our results suggest that Arg145Pro probably impaired cellular autophagy through disrupting the function of CLN5 for incorrect subcellular localization.

## Discussion

NCLs affect all ages and ethnicities, and each subtype is separate disease entity. The main symptoms are cognitive decline, epilepsy, motor deterioration, and visual loss, and the disorder varies and depends on the genetic mutations. The patients with CLN5 diseases develop generally symptoms at the age of 4–7 years, with motor clumsiness or cognition impairment as the initial symptoms (Savukoski et al., 1998). As the disease progressed, visual loss as well as epileptic seizures would be developed. The described patient in this report displayed an initial symptom at 5.5 years, with paroxysmal epilepsy associated with vomiting as initial symptom followed by progressive psychomotor and visual deterioration.

With the advent of new sequencing technologies, the diagnosis of NCL can be made when mutations in pathogenic gene have been identified, in combination with clinical features. Enzyme activity, blood film examination as well as ultrastructural examination of skin remain helpful for confirmation. The diagnosis of NCL for our patient was confirmed by the identification of a homozygous mutation in *CLN5* via whole exome sequencing.

To date, 54 disease associated mutations had been identified in *CLN5* gene. These included 21 missense, 9 nonsense, 11 deletion, 4 insertion mutations, and 9 sequence variants (Xin et al., 2010; Ren et al., 2016; Ge et al., 2018; Zhou et al., 2018; Parvin et al., 2019) (as shown in Table 1). CLN5 has 4 exons and disease-causing mutations are spread through the whole gene, but 36 of 54 variants are located in *CLN5* exon 3 and 4. The nonsense mutation p.Tyr392^∗^, resided in exon 4, is the founder mutation in the Finnish population which resulted in a truncated protein lacking the C-terminal 16 amino acids and accounted for 83% of NCL cases caused by CLN5 defects in Finland (Savukoski et al., 1998). The missense mutation Arg145Pro described in this study is located in exon 2, where 5 pathogenic mutations, including p.Arg112Pro, p.Arg112His, p.Arg112Cys, p.Cys126Tyr and p.Arg145^∗^, has been previously reported. It’s suggested that site 112, 126, and 145 might be mutated hotspots in exon 2 of CLN5.

**TABLE 1:** CLN5 mutation spectrum.

**Case no.**	**Mutation**	**Location**	**Type of mutation**	**Amino acid change**	**Ethnicity**
1	c.225G>A	Exon 1	Nonsense	p.(Trp75*)	Sweden/Canada
2	c.4C>T	Exon 1	Sequence variant	p.(Arg2Cys)	Czech Republic/Turkey
3	c.291insC	Exon 1	Insertion	p.(Ser98Leufs*13)	Argentina
4	c.72A>G	Exon 1	Sequence variant	p.(=)	USA/Argentina
5	c.234C>G	Exon 1	Sequence variant	p.(=)	USA
6	c.61C>T	Exon 1	Missense	p.(Pro21Ser)	Turkey
7	c.223T>C	Exon 1	Missense	p.(Trp75Arg)	Turkey
8	c.334C>T	Exon 2	Missense	p.(Arg112Cys)	China
9	c.335G>A	Exon 2	Missense	p.(Arg112His)	UK
10	c.335G>C	Exon 2	Missense	p.(Arg112Pro)	Portugal
11	c.377G>A	Exon 2	Missense	p.(Cys126Tyr)	USA
12	c.433C>T	Exon 2	Nonsense	p.(Arg145*)	UK
13	c.486+139_712+2132del	Exon 3	Deletion	p.(Lys163Glufs*11)	USA
14	c.524T>G	Exon 3	Nonsense	p.(Leu175*)	Turkey
15	c.527_528insA	Exon 3	Insertion	p.(Gly177Trpfs*10)	Pakistan/USA
16	c.565C>T	Exon 3	Nonsense	p.(Gln189*)	Portugal
17	c.575A>G	Exon 3	Missense	p.(Asn192Ser)	USA
18	c.595C>T	Exon 3	Missense	p.(Arg199*)	China
19	c.528T>G	Exon 3	Sequence variant	p.(=)	Argentina
20	c.593T>C	Exon 3	Missense	p.(Leu198Pro)	Turkey
21	c.613C>T	Exon 3	Missense	p.(Pro205Ser)	Qatar/Yemen
22	c.619T>C	Exon 3	Missense	p.(Trp207Arg)	UK
23	c.620G>C	Exon 3	Missense	p.(Trp207Ser)	China/USA
24	c.623G>A	Exon 3	Missense	p.(Cys208Tyr)	China
25	c.669insC	Exon 3	Insertion	p.(Trp224Leufs*30)	Canada
26	c.671G>A	Exon 3	Nonsense	p.(Trp224*)	UK
27	c.694C>T	Exon 3	Nonsense	p.(Gln232*)	Serbia
28	c.718_719delAT	Exon 3	Deletion	p.(Met240Valfs*13)	China
29	c.726C>A	Exon 4	Missense	p.(Asn242Lys)	Turkey
30	c.741_747delinsTT	Exon 4	Deletion	p.(Trp247Cysfs*5)	Middle East
31	c.741G>A	Exon 4	Nonsense	p.(Trp247*)	Iran
32	c.755-756insC	Exon 4	Insertion	p.(Glu253*)	Sweden
33	c.772T>G	Exon 4	Missense	p.(Tyr258Asp)	Italy
34	c.835G>A	Exon 4	Missense	p.(Asp279Asn)	Portugal/Netherland
35	c.907_1094del188	Exon 4	Deletion	p.(Thr303Cysfs*10)	USA
36	c.919delA	Exon 4	Deletion	p.(Arg307Glufs*29)	Egypt
37	c.935G>A	Exon 4	Missense	p.(Ser312Asn)	Italy
38	c.955_970del16	Exon 4	Deletion	p.(Gly319Phefs*12)	UK
39	c.1026C>A	Exon 4	Nonsense	p.(Tyr342*)	Czech Republic
40	c.1054G>T	Exon 4	Nonsense	p.(Glu352*)	Newfoundland/UK
41	c.1071_1072delCT	Exon 4	Deletion	p.(Leu358Alafs*4)	China/USA
42	c.1072_1073delTT	Exon 4	Deletion	p.(Leu358Alafs*4)	Pakistan/China
43	c.1083delT	Exon 4	Deletion	p.(Phe361Leufs*4)	USA
44	c.1082T>C	Exon 4	Missense	p.(Phe361Ser)	China
45	c.1103_1106delAACA	Exon 4	Deletion	p.(Lys368Serfs*15)	Spain
46	c.1103A>G	Exon 4	Missense	p.(Lys368Arg)	USA
47	c.1121A>G	Exon 4	Missense	p.(Tyr374Cys)	USA
48	c.1137G>T	Exon 4	Missense	p.(Trp379Cys)	Afghanistan
49	c.1175_1176delAT	Exon 4	Deletion	p.(Tyr392*)	Finland
50	c.320+8C>T	Intron 1	Sequence variant	p.(=)	Canada
51	c.320+18C>T	Intron 1	Sequence variant	p.(=)	USA
52	c.321-1G>A	Intron 1	Sequence variant	p.?	China
53	c.486+5G>C	Intron 1	Sequence variant	p.?	Canada
54	c.1224+33A>G	3’UTR	Sequence variant	p.(=)	USA

As listed in Table 1, 24 variants including nonsense, insertion and deletion mutations were supposed to produce a premature termination codon. Nonsense mutation p.Gln189^∗^ was reported to produce a mRNA non-producing allele and reduce CLN5 expression via nonsense-mediated mRNA decay (Bessa et al., 2006), while p.Trp75^∗^ encoded a truncated polypeptide. Both p.Glu253^∗^ and p.Glu352^∗^ produced unstable proteins and increased their recognition and degradation by the ER quality control system (Schmiedt et al., 2010; Larkin et al., 2013). In addition, missense mutations p.Arg112His, p.Arg112Pro, p.Asp279Asn, p.Ser312Asn, and p.Trp379Cys would cause ER retention of the mutant polypeptides, perhaps due to misfolding (Isosomppi et al., 2002; Lebrun et al., 2009; Schmiedt et al., 2010). According to previous reports, approximately half of the pathogenic mutations resulted in prematurely terminated transcripts, impaired subcellular localization of mutated CLN5 for folding defects or reduced protein expression. As for our described missense mutation Arg145Pro, *in silico* predictions by PolyPhen-2, Provean and MutationTaster were damaging, while RaptorX predicted that the change of arginine to proline might greatly interrupt the interaction of Arg145 and other nearby amino acids in space. Based on these predictions, further *in vitro* functional study is needed to clarify the effect of this variant.

For most genetic mutations related to NCLs, complete loss of gene function is due to decreased expression or mislocalization of the encoded protein. Recently, a loss-of-function of CLN5 variant (p.Asn320Ser) was reported to cause endoplasmic reticulum retention, which impaired trafficking of retromer, a master conductor of endosomal sorting and trafficking, and was associated with Alzheimer (Qureshi et al., 2018). In addition, our *in silico* prediction by RaptorX suggested the region, encompassing Phe109∼Val263, formed a structure domain rich in β-sheets. This domain, might have major structural constraints for the function and expression of CLN5, for 12 of 21 missense mutations listed in Table 1 are located in the region. This region may also play a critical role in the correct subcellular localization of CLN5, which also echo previous study that missense mutations Arg112Pro and Arg112His resulted in ER retention of mutated CLN5 (Schmiedt et al., 2010). Thus, we investigated whether Arg145Pro affects protein expression and cellular localization of CLN5. Our experiments showed that mtCLN5 had a comparable expression level with wtCLN5, but little mutated protein could be colocalized with the lysosome marker, LAMP-1, suggesting mutation Arg145Pro affects the intracellular trafficking of CLN5.

Since most of mutated CLN5 could not be targeted to lysosome, the mutation Arg145Pro may be deleterious to the function of CLN5 protein. We attempted to analyze the genotype-phenotype correlations of the loss-of-function mutations and pathogenic mutations with *in vitro* experimental evidence. As listed in Table 2, the majority of CLN5 disease patients presented motor and visual problems as initial symptom. Though pathogenic mutations were located at different regions of CLN5, most patients (18 of 26) presented first symptoms at 3–7 years. Patients carrying homozygous mutation of p.Ser98Leufs^∗^13, p.Gly177Trpfs^∗^10, p.Met240Valfs^∗^13, or p.Trp247^∗^ displayed initial symptoms at 4 months, 5 and 3 years, respectively, while the patient with compound heterzygous mutations of p.Trp75^∗^ and p.Trp224Leufs^∗^30 was onset at 8 years (Cismondi et al., 2008; Xin et al., 2010; Parvin et al., 2019). It seems that there is no obvious relationship between mutation sites and the onset age of CLN5 diseases. In addition, p.Arg112His, p.Asp279Asn, p.Ser312Asn, p.Leu358Alafs^∗^4, p.Trp379Cys or p.Tyr392^∗^ was reported to cause ER retention of mutated polypeptide, the onset age of patients with these homozygous mutations varied from 2 to 56 years (Savukoski et al., 1998; Pineda-Trujillo et al., 2005; Lebrun et al., 2009). It is likely that the impairment of these mutations to function of CLN5 is various, and some of them might retain unknown functions outside lysosome. As previously reported, CLN5 might play a role in intracellular trafficking of proteins by controlling the recycling of lysosomal sorting receptors and CLN5 deficiency was shown to impair the maturation of Cathepsin D encoded by *CLN10*. CLN5 was able to interact with CLN2 and CLN3. Besides, there have been numerous indications of disruption in the autophagy-lysosome pathway in NCL pathogenesis in human and animal models (Thelen et al., 2012; Best et al., 2017). CLN5 deficiency was shown to impair the neurogenesis in knockout mouse model (Savchenko et al., 2017), which was not related to the change in apoptosis but possibly attributed to compromised autophagy (Holmberg et al., 2004). Impaired autophagy was also found in *CLN5^–/–^* ovine neural cultures from sheep with CLN5 disease (Best et al., 2017) and in retinal extracts of mouse models of CLN5 disease (Leinonen et al., 2017). However, the precise function of CLN5 was not well defined and the mechanism underlying the pathogenesis of CLN5 disease remains to be clarified. It’s necessary to further investigate the impacts of pathogenic variants on the function of CLN5, which will help us better understand the clinical heterogeneity of CLN5 disease.

**TABLE 2:** Genotype-phenotype correlation of CLN5 mutations.

**Patient no.**	**Orientation**	**Mutation**	**Impacts on CLN5**	**Country of origin**	**Onset age**	**Initial symptom**
1	Homozygous	p.Ser98Leufs*13	/	Argentina	4M	N.A
2	Homozygous	p.(Arg112His)	ER retention	Colombia	9Y	Visual failure
3	Homozygous	p.(Arg145Pro)	Impairing protein trafficking to lysosome	China	5Y	Paroxysmal epilepsy
4	Homozygous	p.(Gly177Trpfs*10)	/	Pakistan	5Y	Motor difficulty
5	Homozygous	p.(Met240Valfs*13)	/	China	5Y	Mental and motor development retardation
6	Homozygous	p.(Trp247Cysfs*5)	/	Middle East	5Y	NA
7	Homozygous	p.(Trp247*)	/	Iran	3Y	Ataxia and myoclonus
8	Homozygous	p.(Asp279Asn)	ER retention	Netherland	2Y	NA
9	Homozygous	p.(Arg307Glufs*29)	/	Egypt	6Y	Motor difficulty
10	Homozygous	p.(Ser312Asn)	Affecting ER-lysosome protein trafficking	Italy	50-56Y	Unsteady gait
11	Homozygous	p.(Tyr342*)	/	Czech Republic	5.5Y	NA
12	Homozygous	p.(Glu352*)	Unstable protein	UK	5~6.6Y	NA
13	Homozygous	p.(Leu358Alafs*4)	ER retention	Pakistan	4-5Y	NA
14	Homozygous	p.Lys368Serfs*15	/	Spain	7Y	NA
15	Homozygous	p.(Trp379Cys)	ER retention	Afghanistan	2~3y	NA
16	Homozygous	p.(Tyr392*)	ER retention, destabilized polypeptide	Finland	3~4Y	NA
17	Homozygous	c.320+8C>T, p.(=)	/	Canada	1Y	NA
18	Compound heterozygous	p.(Arg112Pro)	ER retention	Portugal	3Y	Attention deficits and speech regression
		p.Gln189*	Resulting in a mRNA non-producing allele			
19	Compound heterozygous	p.Trp224*	/	USA	5Y	Motor difficulty andvisual loss
		p.Lys368Serfs*15	/			
20	Compound heterozygous	p.Trp224*	/	USA	5Y	Seizures
		p.Thr303Cysfs*10	/			
21	Compound heterozygous	p.Tyr374Cys	Protein destabilization	USA	17Y	Cognitive regressionand visual loss
		p.Cys126Tyr	/			
22	Compound heterozygous	p.Tyr374Cys	Protein destabilization	USA	17Y	Motor difficulty
		p.Thr303Cysfs*10	/			
23	Compound heterozygous	p.Trp75*	Encoding a truncated polypeptide	Sweden	8Y	Motor difficulty
		p.Trp224Leufs*30	/			
24	Compound heterozygous	p.Leu358Alafs*4	/	China	4Y	Visual loss
		p.Trp207Ser	/			
25	Compound heterozygous	p.Tyr392*	ER retention, destabilized polypeptide	Finland	4Y	NA
		p.Trp224Leufs*30	/			

*The slash (/) indicates the mutation lacks of functional study in vitro; NA means not available.*

In conclusion, as patients with NCLs have a similar clinical manifestation that seems not directly linked to the specific causative genes, whole exome sequencing facilitates the diagnosis by identifying the pathogenic gene with a single test. In order to determine causality, *in silico* tools and *in vitro* functional studies are needed to perform to provide evidence for the pathogenic nature of each mutation identified. Impaired protein stability and intracellular trafficking could be important factor associated with the pathogenesis of CLN5 disease in most NCLs patients. Our study reported a deleterious effect of the variant discovered in our patient on CLN5 by affecting intracellular trafficking, leading to the NCL disease and emphasizes the usefulness of next generation sequencing in molecular diagnosis.

## Data Availability Statement

The datasets generated for this study are available on request to the corresponding author.

## Ethics Statement

The studies involving human participants were reviewed and approved by the Wuhan Children’s Hospital Ethics Committee. Written informed consent was obtained from the patients’ legal guardian/next of kin for the publication of any potentially identifiable images or data included in this article.

## Author Contributions

XH, AZ, and GW contributed to the conception and design of the study. SL did the experiments and wrote the first draft of the manuscript. BB provided the clinical information of the patient. BZ, LT, and PZ wrote the sections of the manuscript. All authors contributed to manuscript revision, read and approved the submitted version.

## Conflict of Interest

The authors declare that the research was conducted in the absence of any commercial or financial relationships that could be construed as a potential conflict of interest.

## Footnotes

1 http://www.hgmd.cf.ac.uk/

2 http://genetics.bwh.harvard.edu/pph/

3 http://www.Blocks.fhcrc.org/sift/SIFT.html

4 http://www.mutationtaster.org/

5 https://cadd.gs.washington.edu/score

6 http://raptorx.uchicago.edu/

7 http://www.ucl.ac.uk/ncl
